# Synaptotagmin 13 is neuroprotective across motor neuron diseases

**DOI:** 10.1007/s00401-020-02133-x

**Published:** 2020-02-17

**Authors:** M. Nizzardo, M. Taiana, F. Rizzo, J. Aguila Benitez, J. Nijssen, I. Allodi, V. Melzi, N. Bresolin, G. P. Comi, E. Hedlund, S. Corti

**Affiliations:** 1 Dino Ferrari Centre, Neuroscience Section, Department of Pathophysiology and Transplantation (DEPT), University of Milan, Milan, Italy; 2 Foundation IRCCS Ca’ Granda Ospedale Maggiore Policlinico, Neurology Unit, Via Francesco Sforza 35, 20122 Milan, Italy; 3 Department of Neuroscience, Biomedicum D7, Karolinska Institutet, Solna v. 9, 171 77 Stockholm, Sweden; 4 Foundation IRCCS Ca’ Granda Ospedale Maggiore Policlinico, Neuromuscular and Rare Diseases Unit, Via Francesco Sforza 35, 20122 Milan, Italy

## Abstract

In amyotrophic lateral sclerosis (ALS) and spinal muscular atrophy (SMA), spinal and lower brainstem motor neurons degenerate, but some motor neuron subtypes are spared, including oculomotor neurons (OMNs). The mechanisms responsible for this selective degeneration are largely unknown, but the molecular signatures of resistant and vulnerable motor neurons are distinct and offer clues to neuronal resilience and susceptibility. Here, we demonstrate that healthy OMNs preferentially express *Synaptotagmin 13 (SYT13)* compared to spinal motor neurons. In end-stage ALS patients, *SYT13* is enriched in both OMNs and the remaining relatively resilient spinal motor neurons compared to controls. Overexpression of SYT13 in ALS and SMA patient motor neurons in vitro improves their survival and increases axon lengths. Gene therapy with *Syt13* prolongs the lifespan of ALS mice by 14% and SMA mice by 50% by preserving motor neurons and delaying muscle denervation. SYT13 decreases endoplasmic reticulum stress and apoptosis of motor neurons, both in vitro and in vivo. Thus, SYT13 is a resilience factor that can protect motor neurons and a candidate therapeutic target across motor neuron diseases.

**Electronic supplementary material:**

The online version of this article (10.1007/s00401-020-02133-x) contains supplementary material, which is available to authorized users.

## Introduction

Amyotrophic lateral sclerosis (ALS) and spinal muscular atrophy (SMA) are lethal neurodegenerative diseases characterized by a progressive loss of motor neurons in the spinal cord, brainstem, and cortex [9, 21]. However, some motor neurons are preserved throughout late stages of these diseases, including oculomotor neurons (OMNs), trochlear neurons and neurons in the abducens, which regulate eye movement as well as Onuf's nuclei, which controls sphincter function. This has been demonstrated in both mouse models [11, 24, 29, 32, 55] and in *post-mortem* tissues from patients [31, 35, 39, 60]. Notably, both the familial (*f*) and sporadic (*s*) forms of ALS and SMA share this pattern of selective motor neuron resistance [35, 61]. Consequently, eye movement and sphincter function remain relatively preserved, even in the advanced stages of these diseases and ocular tracking have high utility for communication [35, 64]. This preservation across diseases indicates that differential vulnerability between motor neuron groups is largely independent on the cause of disease, and that mechanisms of vulnerability and resilience could be shared across diseases [9–11, 20]. Elucidation of the molecular basis of selective resistance may lead to the development of new therapies to prevent the relentless motor neuron loss. Previous studies of ALS and SMA [52] suggest that elements intrinsic to motor neurons are critical to the initiation and early progression of degeneration [6, 62]. We and others previously examined the gene expression patterns of vulnerable and resistant motor neuron groups and identified molecular differences that may account for their observed differential vulnerability [1, 4, 5, 7, 10, 29, 33, 36, 56, 68].

To identify additional candidates of motor neuron resistance, we conducted a careful bioinformatics analysis of two previously published microarray data sets on isolated OMNs and spinal motor neurons in the rat and mouse. We identified 24 genes, including *synaptotagmin 13* (*SYT13*), with common preferential expression in OMNs across species (mouse and rat) and ages (1 week and 8 weeks of age). RNA scope analysis in human control *post-mortem* tissues confirmed the localization and preferential expression of *SYT13* in OMNs compared to spinal motor neurons also in man. RNA-seq analysis of motor neurons in end-stage ALS patient tissues demonstrated *SYT13* enrichment in the remaining resilient neurons in both oculomotor nucleus and spinal cord compared to controls.

SYT13 belongs to a family of synaptotagmins (SYTs) that are vesicular trafficking proteins important for synapsis and vesicle metabolism [65]. Unlike many other synaptotagmins, SYT13 binds to cellular membranes in a Ca^2+^-independent fashion [22]. Knowledge of SYT13 function is very limited, but SYT13 has been hypothesized to be involved in general vesicle trafficking and synaptic vesicle docking, facilitation of membrane fusion and exocytosis, and interactions with neurexin1 [22]. Based on the strong OMN expression of SYT13 in healthy controls, and its preferential expression in all remaining relatively resilient motor neurons in ALS patient tissues, as well as its functional implications in processes related to ALS and SMA, we pursued SYT13 in the context of motor neuron diseases.

We investigated the effect of SYT13 on motor neurons from ALS and SMA patients and in transgenic mouse models of ALS and SMA. Notably, we found that up-regulation of SYT13 induces motor neuron protection in vitro and in vivo, prolonging the lifespan of both ALS and SMA mice, while decreasing apoptosis and ER stress and improving axon growth. Thus, our approach of using degeneration-resistant OMNs as a tool to identify motor neuron-protective molecules was validated and identified SYT13 as a candidate therapeutic target for motor neuron diseases.

## Materials and methods

### Ethics statement

The studies involving human or animal tissues were conducted in compliance with the Code of Ethics of the World Medical Association (Declaration of Helsinki) and with national legislation and institutional guidelines. All animal experiments were reviewed and received approval by the Italian Ministry of Health and Swedish animal ethical (Stockholms Norra Djurförsöksetiska nämnd) review boards. Ethical approval for the use of the human *post-mortem* specimens was granted from the regional ethical review board in Stockholm, Sweden (Regionala Etikprövningsnämnden, Stockholm, EPN). Human CNS samples were obtained from the Netherlands Brain Bank (NBB, www.brainbank.nl) and the National Disease Research Interchange (NDRI, www.ndriresource.org) with the written informed consent from the donors or next of kin (Table Supplementary 1, online resource). Human fibroblast cell lines were obtained from Eurobiobank with informed consent (ethical committee approved at the IRCCS Foundation Ca' Granda Ospedale Maggiore Policlinico, Table Supplementary 2, online resource).

### RNA scope staining of human tissues

To confirm the RNA-seq expression of SYT13, we used RNA scope [70]. RNA scope is a novel RNA in situ hybridization technology with a unique probe design (double-Z design) that allows simultaneous signal amplification and background suppression to achieve single-molecule visualization. The technique is compatible with formalin-fixed, paraffin-embedded tissue specimens and can use conventional chromogenic dyes for brightfield microscopy or fluorescent dyes. Fresh-frozen midbrain or spinal cord samples from six controls subjects (Supplementary Table 1, online resource) were sectioned at 10 µm thickness in poly-l-lysine coated glass slides (Sigma, P0425) and kept at − 80 °C. Prior to staining, slides were quickly thawed and fixed with fresh PFA (4% in PBS) for 1 h at 4 °C. The RNA scope 2.5 HD Assay—RED Kit (Cat. 322360) was used using manufacturer recommendations.

To set up the procedure, we tested a negative control probe against a bacterial gene (Cat. 310043, dapB-C1) and two different positive control probes against *peptidylprolyl isomerase b* (Cat No. 313901, PPIB-C1) and the *vesicular acetylcholine transporter-member A3* (Cat No. 519321, VACHT-C1), (Supplementary Fig. 1, online resource).

Once the assay was in place, sections were stained with a probe against *Synaptotagmin 13* (Cat No. 552271, SYT13-C1). Slides were Nissl-counterstained with fresh 50% Gill No. 1 solution (Cat. GSH132-1L, Sigma-Aldrich) for 4 min, washed in water and dried for 15 min at 60 °C before mounting with Pertex (Cat. 00811, Histolab).

For every sample (*N* = 3, for OMNs and spinal cord (SC) motor neurons), we imaged up to 30 random fields within the OMN region or the ventral horn in the spinal cord. Pictures were captured at 40× magnification using the bright field of a Leica microscope (DM6000/CTR6500 and DFC310 FX camera). After randomization and decoding of all the images, cells were scored in a blinded fashion using a scale from 0 to 4, based on the number of dots (SYT13-RNA clouds) per cell using ImageJ (version 1.48). Around 75 cells were scored per subject and region (OMN: 74.17 ± 36.39 and SC: 74.50 ± 23.43) following the scoring guidelines from Advanced Cell Diagnostics (ACD, RNA scope 2.5 HD Assay—RED Kit User Manual page 16). In brief, the scoring was as follows: Score 0: No staining or less than 1 dot every 10 cells. Score 1: 1–3 dots/cell. Score 2: 4–10 dots/cells, very few dot clusters. Score 3: > 10 dots/cell with < 10% of positive cells having dot clusters, and Score 4: > 10 dots/cell with > 10% of cells showing dot clusters. Investigators performing the quantifications were blinded to the target region (OMN and SC).

### RNA sequencing of human OMNs and spinal cord motor neurons isolated by laser capture microscopy

As motor neurons are easily identifiable by their distinct location in the oculomotor nucleus in the midbrain and ventral horn of the spinal cord and by their large soma sizes, human CNS tissues were subjected to a quick histological (Nissl) staining based on the Arcturus Histogene Staining Kit protocol. Slides were placed into the slide holder of the microscope (Leica DM6000R/CTR6500) and captured using the Leica LMD7000 system. To avoid contamination by surrounding cells, cutting outlines were drawn closely around individual motor neurons. cDNA library preparation for sequencing on an Illumina HiSeq2000 sequencer was carried out with a slightly modified version of the Smart-seq2 protocol [53, 54]. RNA-seq reads were mapped to the human reference genome hg38/GRCh38 (Ensembl version 81) using STAR [17] (version 2.4.1). To visualize the expression of genes/transcripts, Cufflinks [66] (version 2.2.1) was used to generate RPKM values with the parameter library-norm-method set as *geometric*. Quality control was conducted and samples with lower than 1 million reads or < 70% mapping ratio to the genome or < 7000 genes expressed ≥ 1 RPKM were removed from further analysis. Differential gene expression analysis was performed on the read counts using DESeq2 [38]. Only genes with counts in a minimum of two samples were included for analysis. A gene was considered differentially expressed at an adjusted *P* < 0.05.

### RNA-seq data availability

Fourteen new LCM-seq samples (corresponding to OMNs or spinal motor neurons from ALS patients) included in this study have been deposited in the GEO database under accession number GSE115130. Six control SC samples were previously submitted under the GEO accession GSE76514 [46] and an additional 32 control samples (both OMN and SC) were also published by our group under accession GSE93939 [2] (Supplementary Table 1, online resource).

### Animal models

This study used adult male and female SOD1^G93A^ mice (B6.Cg-Tg(SOD1-G93A)1Gur/J [28] as a model of fALS and non-transgenic littermates served as controls. This model shows early symptoms at 80 days of age. Mice were genotyped as previously described [47]. The triple-mutant SMA∆7 (FVB.Cg-Tg(SMN2*delta7)4299Ahmb Tg(SMN2)89Ahmb Smn1tm1Msd) transgenic model [37] was used as an in vivo model of SMA (male and female). The delta7 mutation refers to an artificial SMN2 cDNA that lacks exon 7, that is added to the Smn knockout mice that already express SMN2, to further extend the animal survival from 5–7 days to 15 days, thus making the mouse models more suitable for translational experiments. In particular, the Tg(SMN2*delta7)4299Ahmb allele is an SMA cDNA lacking exon 7, while the Tg(SMN2)89Ahmb allele is the entire human SMN2 gene. Heterozygous Smn knockout mice with the SMN2 transgenes were bred to obtain homozygous mice for the knockout Smn alleles (SMA mice, SMN2 + / + ;SmnΔ7 + / + ;mSmn − / −) and pups were identified by genotyping [37] (protocol approval 1007/2016 PR by Italian Ministry of Health). All animals were kept according to standard conditions, including access of food and water *at libitum* and dark/light cycle of 12 h.

### Immunohistochemistry in rodent and human tissues

*Immunohistochemistry of murine tissues:* mice were euthanized at 120 days of age (SOD1) or at P10 (SMA). Tissues were fixed in 4% paraformaldehyde for 24 h followed by 20% sucrose solution overnight and frozen in liquid nitrogen-cooled isopentane [2, 47]. Tissues were cryosectioned and mounted on gelatinized glass slides. Every 10th section (20 µm) was collected and analysed. For immunohistochemistry, all sections were saturated with 10% bovine serum albumin and 0.3% Triton X-100 for 1 h at room temperature before incubation with primary antibodies overnight at 4 °C (see Supplementary Table 3, online resource for primary antibodies). The day after, slides were incubated with Alexa Fluor secondary antibodies (1:1000; Life Technologies). Lumbar spinal cord segments L3–L5 for ALS mice and L1–L2 for SMA mice (*N* = 3 mice/group) were stained with NeuroTrace 435/455 Blue Fluorescent Nissl Stain (1:100, Life Technologies), incubated for 1 h. Intercostal muscles for SMA mice and tibial anterior (TA) muscles for SOD1 animals (*N* = 6 mice/group) were stained for presynaptic neurofilament medium (NFM, 1:250) and post-synaptic α-bungarotoxin 555 (BTX, 1: 200); LEICA LCS6 confocal microscope was used. Negative controls were performed for all stainings.

For in vivo motor neuron quantification, lumbar spinal cord slices were stained with Nissl (conventional histology). Only neurons with an area ≥ 80 μm^2^ [14, 69] and located in a position congruent with that of motor neuron groups were counted on serial sections under transmitted light microscopy [47]. For NMJ quantification, a minimum of 100 NMJs from each muscle/group were randomly selected and assessed under the microscope to determinate the number of denervated or degenerated junctions based on co-localization of the two antibody signals.

*Immunohistochemistry of human tissues:* characteristics of non-demented (ND) control *post-mortem* tissues used for immunohistochemical analysis are described in Supplementary Table 1, online resource. Tissues from NBB were embedded in paraffin and were sectioned (10 µm) on a sliding microtome. Tissues from NDRI were fixed in PFA, sequentially placed through sucrose gradients for cryoprotection and sectioned (40 µm) on a freezing microtome. NBB tissue was deparaffinized and all tissues underwent antigen retrieval (0.01 M citric acid buffer, pH 6.0 for 20 min at 95 °C) and blocking of endogenous peroxidases (3% H_2_O_2_ in 50% methanol in PBS) prior to staining. Tissues were blocked and stained with primary antibodies for 2 days (Supplementary Table 3, online resource). Tissue was washed and incubated with a biotinylated secondary antibody (1:100; Vector Laboratories) for 3 h, with subsequent incubation in streptavidin–biotin complex (Vectastain ABC kit Elite, Vector laboratories) for 1 h and visualized by incubation in 3,3′-diaminobenzidine solution (DAB, Vector Laboratories). Myers haematoxylin (Histolab) was utilized for counterstaining; tissue was dehydrated by sequential steps in increasing ethanol concentration and coverslipped using Mountex (Histolab). Brightfield images were captured using a Zeiss Axio Imager M1 Upright microscope.

### iPSC lines’ generation and motor neuron cultures

*Differentiation of human iPSCs into motor neurons*: iPSC lines were reprogrammed from human fibroblasts as described in Supplementary Table 2, online resource. The cells were tested for Mycoplasma (MycoAlert kit, Lonza). ALS, SMA, and control iPSCs were differentiated into motor neurons using a multistep protocol, based on embryoid body formation and addition of specific cytokines, already described in the literature [42]. To monitor the proper acquisition of a motor neuron phenotype, cells were transduced with a lenti-Hb9*::GFP* construct [12, 40], fixed and stained for quantification utilizing established neuronal and motor neurons markers. Motor neuron cultures were transduced with a lentivirus-*SYT13* or a *null* vector as the negative control [63].

*Induction of ALS-like toxicity*: To model ALS conditions in vitro, iPSC-derived motor neurons were either co-cultured with toxic murine SOD1^G93A^ astrocytes [16, 40] or exposed to glutamate excitotoxicity [18, 29]. For the SOD1^G93A^ astrocyte toxicity assay, iPSCs-derived motor neurons were cultured on the bottom compartment of a transwell co-culture system in the presence of astrocytes (in the upper compartment) obtained either from SOD1^G93A^ mice or WT mice. The porous membrane that separates the two compartments only allows diffusion of soluble molecules. Glial cells were obtained from embryonic spinal cords as previously described [2, 43] and seeded at 20,000 cells per cm^2^ [43]. To perform the excitotoxicity assay, motor neurons were maintained for 7 days in a neurotrophin-deprived medium prior to the glutamate supplementation. Toxicity was induced by administration of 20 µM glutamate and 100 µM L-trans-Pyrrolidine-2,4-dicarboxylic acid (PDC) [2, 29]. Glutamate excitotoxicity was induced for 7 days, while the astrocyte co-culture assay lasted 3 weeks. Motor neurons were counted at the end of the toxic stimuli.

*SMA-like degeneration of human motor neurons:* SMA patient-specific motor neurons derived from iPSCs present a cell-autonomous degeneration when maintained long-term in vitro (8 weeks) [13, 19, 48], allowing for SMA disease modelling. Motor neurons were counted at the end of the 8 weeks.

*Immunocytochemistry and quantification of motor neuron cultures:* Cells were fixed in 4% paraformaldehyde for 10 min, blocked with 10% bovine serum albumin and 0.3% Triton X-100 in 1X PBS for 1 h at room temperature. Samples were then incubated overnight in primary antibodies at 4 °C as described in Table S3. After washing, secondary antibodies were applied for 2 h at room temperature (Alexa Fluor 488 and Alexa Fluor 594, 1:500; Life Technologies). The anti-SYT13 antibody was visualized utilizing biotin-streptavidin amplification (anti-rabbit biotinylated antibody, 1:400, DAKO; Cy3-streptavidin,1: 500, Sigma). Negative controls were performed for all stainings. Microphotographs were taken with a LEICA LCS2 confocal microscope.

Motor neurons were quantified by selecting 10 random fields for each sample and counting the number of HB9 + cells. Morphometric and axonal length analyses were performed by measuring soma diameter and length distance between two points (one point from the soma and one on the distal axon) [13]. All analyses were carried out in double-blind fashion.

*ER calcium analysis and imaging:* Cells loaded with 5 μM Fluo4 AM FLUO4 were acquired using a Zeiss Axio Observer.Z1 (Carl Zeiss, Jena—Germany) equipped with Hamamatsu EMCCD 9100-02 (Hamamatsu Photonics, Japan) and with a Zeiss FLUAR 40X oil immersion objective (NA: 1.3). Exposure time was set to 50 ms and images were acquired every 5 s for at least 5 min. Images were collected at 14-bit depth and binning 2 (pixels resolution 500 × 500) and analysed using ImageJ (NIH, Maryland—USA). Calcium variations were assessed by fluorescence measurements upon application of 20 µM thapsigargin in PBS depleted of CaCl_2_ and MgCl_2_ and calculated as changes in fluorescence intensity occurring in cell bodies as a fold increase above the normalized baseline (ΔF/F0).

### Western blot analysis of iPSC-derived motor neurons

Western blot analysis was performed as previously described [50]. Briefly, cells were sonicated on ice for 10 min in buffer supplemented with a protease and phosphatase inhibitor cocktail (Pierce) as described [13]. An amount of 60 µg was separated on a 10% sodium dodecyl sulfate–polyacrylamide gel electrophoresis SDS–PAGE. Proteins were transferred on a nitrocellulose membrane and incubated with primary antibodies (Table Supplementary 3, online resource) overnight at 4 °C. Blots were then incubated in secondary antibodies (polyclonal goat anti-rabbit HRP 1:1000, Dako or 1:2700, Life Technologies) and the immune complexes were revealed with a chemiluminescence assay (Amersham). The nitrocellulose membrane was stripped and re-probed with an anti-actin antibody (1:1000) as the loading control.

### Virus administration, behavioural and survival analysis in ALS and SMA animal models

A total dosage of 11 × 10^11^ particles of AAV9 vector expressing *Syt13* or *Gfp* was administered bilaterally into the hindlimb quadriceps and the thoracic muscles of SOD1^G93A^ animals at 80 days of age (*n* = 12 for AAV9*::Syt13*, *n* = 5 for AAV9*::GFP*). AAV9::*null* served as a control vector (*n* = 15). The same muscles were injected bilaterally also in SMA mice with a total dosage of 5 × 10^10^ particles. Here, injections were performed at P1 (*n* = 10 for AAV9*::Syt13* and *n* = 10 for AAV9::*null*). Animals were randomized using an assigned animal identification number. Power analysis using GraphPad was performed to calculate the number of mice needed to treat to detect a difference of 10% in lifespan with 80% power (*β* = 0.8) at a significance level of 0.05. Disease onset, progression, survival, weight and motor performance were monitored after AAV9*::Syt13* or AAV9*::null* treatment. All tests were performed blinded to the mouse genotype and treatment. Motor functions were analyzed utilizing the righting test for SMA animals and the inverted grid assay for the SOD1^G93A^. Mice were sacrificed when unable to right themselves within 30 s when placed on either side [50].

### Statistical analyses

Statistical analyses were carried out utilizing StatsDirect for Windows (version 2.6.4) or GraphPad Prism 5 software. Multiple comparisons on a single data set were performed with one-way analysis of variance (ANOVA), and when several variables were taken into account, the two-way ANOVA was used, followed by appropriate post hoc analysis. Two-tailed, unpaired Student's *t* test was employed to compare two groups. Differences in axonal length were investigated by the Kolmogorov–Smirnov test (https://www.physics.csbsju.edu/stats/KS-test.n.plot_form.html). Kaplan–Meier log rank test and logistic regression analysis were used to compare lifespan and righting test, respectively. Chi-square test was used to determine statistical differences in NMJ innervation. All experiments were carried out at least in triplicate. The experimental results are shown as mean ± SEM or mean ± SD. The null hypothesis was rejected at the 0.05 level.

## Results

### SYT13 is enriched in resistant motor neurons in healthy controls and ALS patients

**Fig. 1:**
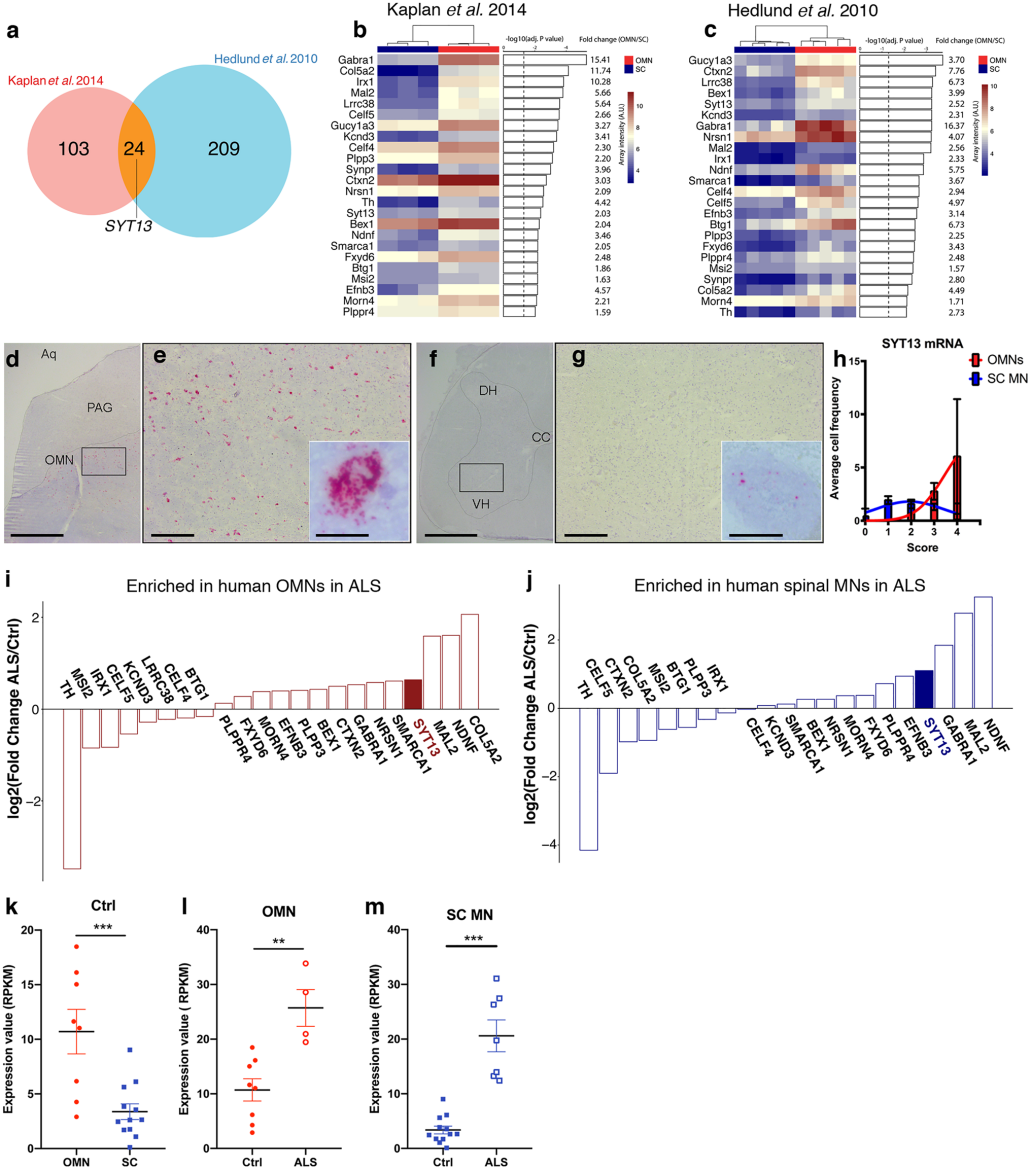
SYT13 is enriched in resistant motor neurons in controls and ALS patients. **a** Venn diagram of the overlap in genes with higher expression in oculomotor neurons (OMNs) compared to spinal motor neurons (MNs) across two microarray studies conducted in adult rats [29] and P7 mice [33]. A total of 24 genes were preferential to OMNs across the two studies. **b–c** Heat maps of the 24 differentially expressed genes with preference for OMNs including adjusted *P* values and fold change. **d–h** RNA scope of human *post-mortem* tissues shows **d, e** the abundance of *SYT13* mRNA in OMNs and **f, g** lower expression in spinal cord MNs, as quantified in (**h**). **i, j** RNA sequencing of human OMNs and spinal MNs from control and ALS patient tissues and analysis of the previously identified 24 genes with enriched expression in OMNs shows that **i***SYT13* is one of the most enriched transcripts in ALS OMNs, and that **j***SYT13* is enriched in the remaining spinal MNs in ALS patient tissues compared to control tissues. Analysis of RNA sequencing data shows higher expression of *SYT13* in OMNs compared to spinal MNs in control individuals (**k**, ***P < 0.001) and demonstrates that *SYT13* mRNA is induced in resistant OMNs in ALS (**l**, **P < 0.01). In addition, *SYT13* is more highly expressed in the remaining relatively resilient spinal MNs in ALS compared to control spinal MNs (**m,** ***P < 0.001). Scale bars = 2000 μm in **d, f**, 400 μm in **e, g** (30 μm in the insets)

To further our understanding of neuronal vulnerability and resilience in ALS, we conducted a careful bioinformatics analysis comparing two microarray data sets on OMNs (resilient in ALS) and spinal motor neurons (vulnerable in ALS) from adult (8 week old) rats [30] and P7 mice [33]. We identified 24 differentially expressed genes (DEGs) commonly enriched in OMNs across the two studies (Fig. 1a). SYT13 was among these 24 DEGs with strong preferential expression in OMNs (Fig. 1a–c), which we found particularly interesting, because SYTs are vesicular trafficking proteins important for synapses. To follow-up on this finding, we conducted RNA scope on human control *post-mortem* tissues from midbrain and spinal cord using a probe against human *SYT13* to confirm the cellular localization of *SYT13* mRNA in OMNs and to quantify the levels at the single-molecule level. We counterstained with Nissl to identify motor neurons based on size and location. To validate the method, we used one negative control probe against the bacterial gene *dihydrodipicolinate* (*dapB*), which, as expected, did not give any signal in human tissues (Supplementary Fig. 1a,b). We also used a general positive control probe against the enzyme peptidylprolyl *cis*–*trans* isomerase B (PPIB), which, as anticipated, gave positive signals in all cells, including motor neurons (Supplementary Fig. 1c, d, online resource), and a probe against vesicular acetylcholine transporter (VACHT), which is specifically present in motor neurons and gave the expected signal (Supplementary Fig. 1e, f, online resource). Using the probe specific for *SYT13,* we found that *SYT13* mRNA was highly expressed also in human OMNs (Fig. 1d, e, h), with lower levels in spinal motor neurons (Fig. 1f–h), validating the transcriptome data from rodents and showing the specificity of *SYT13* expression in human OMNs. We reasoned that, for *SYT13* to be relevant to the preservation of particular motor neurons throughout the detrimental ALS disease process, the transcript should be maintained or elevated in end-stage ALS. To investigate whether this was the case, we conducted laser capture microdissection coupled with polyA-based RNA sequencing (LCM-seq) [45, 46] on pools of individually isolated OMNs and spinal motor neurons from control and ALS patient tissues. Quality analysis of the sequenced neurons revealed that neuronal markers, including neurofilaments, tubulins, peripherin, and *VACHT*, were expressed at high levels across OMN and spinal motor neuron samples (Supplementary Fig. 1g, online resource). Glial markers were present, but at very low levels, and markers of oligodendrocytes and microglia were absent in the majority of samples (Supplementary Fig. 1g, online resource). Principal component analysis (PCA) based on all genes expressed at > 1 RPKM in five samples or more, separated OMNs and spinal motor neurons on the PC2 axis (Supplementary Fig. 1h, online resource). Analysis of *HOX* and *PHOX* gene expression clustered OMNs away from spinal motor neurons based on their distinct anterior–posterior positions in the nervous system (Supplementary Fig. 2i, online resource). Notably, the human LCM-seq data showed that *SYT13* mRNA was expressed at higher levels in OMNs than spinal motor neurons in control tissues (Fig. 1k, *P* < 0.001), confirming our RNA scope data. In-depth bioinformatics analysis of the 24 DEGs enriched in rodent OMNs (Fig. 1a–c) showed that SYT13 was enriched in human OMNs in ALS compared to controls (Fig. 1i, l, *P* < 0.01). Notably, the remaining relatively resilient spinal motor neurons in ALS patient tissues also expressed significantly higher levels of *SYT13* than motor neurons in general in control spinal cords (Fig. 1j, m, *P* < 0.001). Immunohistochemistry of SYT13 protein in human *post-mortem* tissues (Supplementary Table 1, online resource) demonstrated its presence in both OMNs and spinal motor neurons, with the staining in OMNs appearing more distinct (Supplementary Fig. 1j, k, online resource). Taken together, these data clearly show that *SYT13* expression is preferential to resistant OMNs, as well as relatively resilient spinal motor neurons remaining in end-stage ALS patient tissues, suggesting that SYT13 could play a beneficial role in these cells.

### SYT13 protects vulnerable ALS and SMA patient-derived motor neurons

**Fig. 2:**
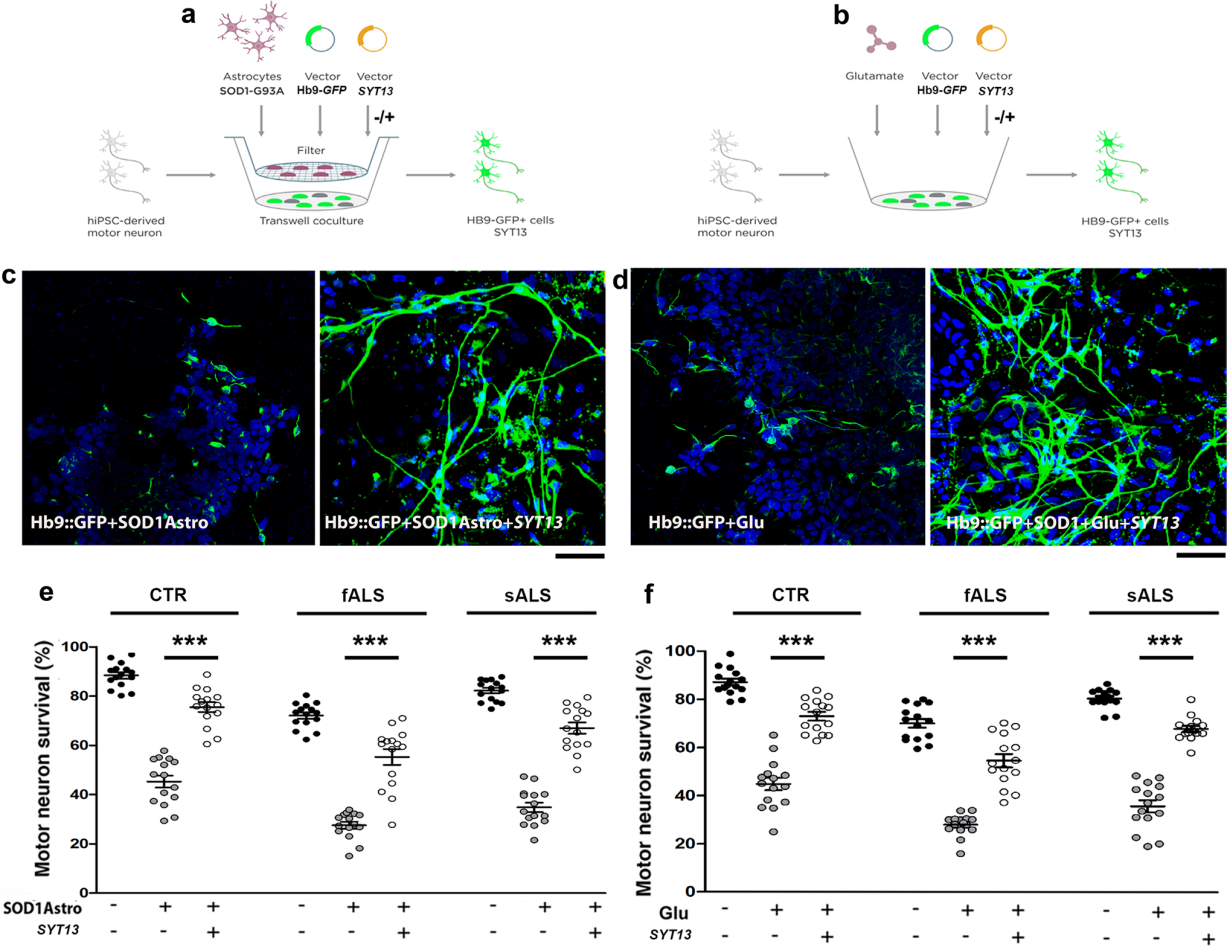
SYT13 protects human spinal motor neurons from ALS-like toxicity in vitro. (**a, b**) To model ALS disease, human spinal motor neurons (MNs) from healthy subjects (CTR) and sALS/fALS patients were exposed to toxic stimuli, comprising three weeks of co-culture with SOD1^G93A^ astrocytes (**a)** or 7 days of glutamate overload (**b**). **c, d** Representative images of ALS motor neurons (Hb9::GFP, green, DAPI, blue signal). The motor neurons were progressively lost after co-culture with SOD1^G93A^ astrocytes, whereas overexpression of SYT13 could rescue motor neuron survival (**c, e**; ***P < 0.0001, *F*(8,126)  = 116.1; one-way ANOVA). Exposure of cultures to glutamate also induced motor neuron degeneration, which could be rescued by overexpression of SYT13 (**d, f**; ***P < 0.0001, *F*(8,126) = 113.8; one-way ANOVA). Motor neurons positive for HB9 and SYT13 were counted at the end of the toxic stimuli. Values are presented as means ± SEM from five independent experiments, three samples/group. Scale bar = 75 µm

Next, we investigated whether up-regulation of SYT13 could halt the degeneration of ALS and SMA patient motor neurons derived from induced pluripotent stem cells (iPSCs) (Supplementary Fig. S2a, online resource). The in vitro differentiation protocol gives rise to 80% motor neurons with a near absence of progenitor cells and proliferating cells, as few cells were positive for progenitor markers OLIG2 and PAX6 and proliferation marker KI67, in line with the literature [42]. Motor neurons generated from human iPSCs, which were monitored by a lenti-Hb9*::eGFP* reporter construct, appeared healthy, had elongated neurites (Supplementary Fig. 2b, online resource) and demonstrated a significant increase in SYT13 mRNA and protein after transduction with a vector encoding the human *SYT13* cDNA (Supplementary Fig. 2c, d, online resource, *P* < 0.001 and *P* < 0.05, respectively). To mimic non-cell-autonomous ALS degeneration in vitro, we used two different assays of progressive motor neuron death: co-culture with toxic SOD1^G93A^ astrocytes or glutamate-induced excitotoxicity [2] (Fig. 2a, b). ALS and control motor neurons degenerated in response to either co-culture with SOD1^G93A^ astrocytes or glutamate excitotoxicity (Fig. 2c, d, left panel). Overexpression of *SYT13* rendered motor neurons more resilient to ALS toxicity (*P* < 0.0001; Fig. 2c–f). We performed the same experiments in C9ORF72 iPSC-derived motor neurons (Supplementary Table 2, online resource). The overexpression of SYT13 significantly increased survival (Supplementary Fig. 3, online resource*, P* < 0.01, two lines, three independent experiments/line), in line with the other ALS motor neuron lines tested.

**Fig. 3:**
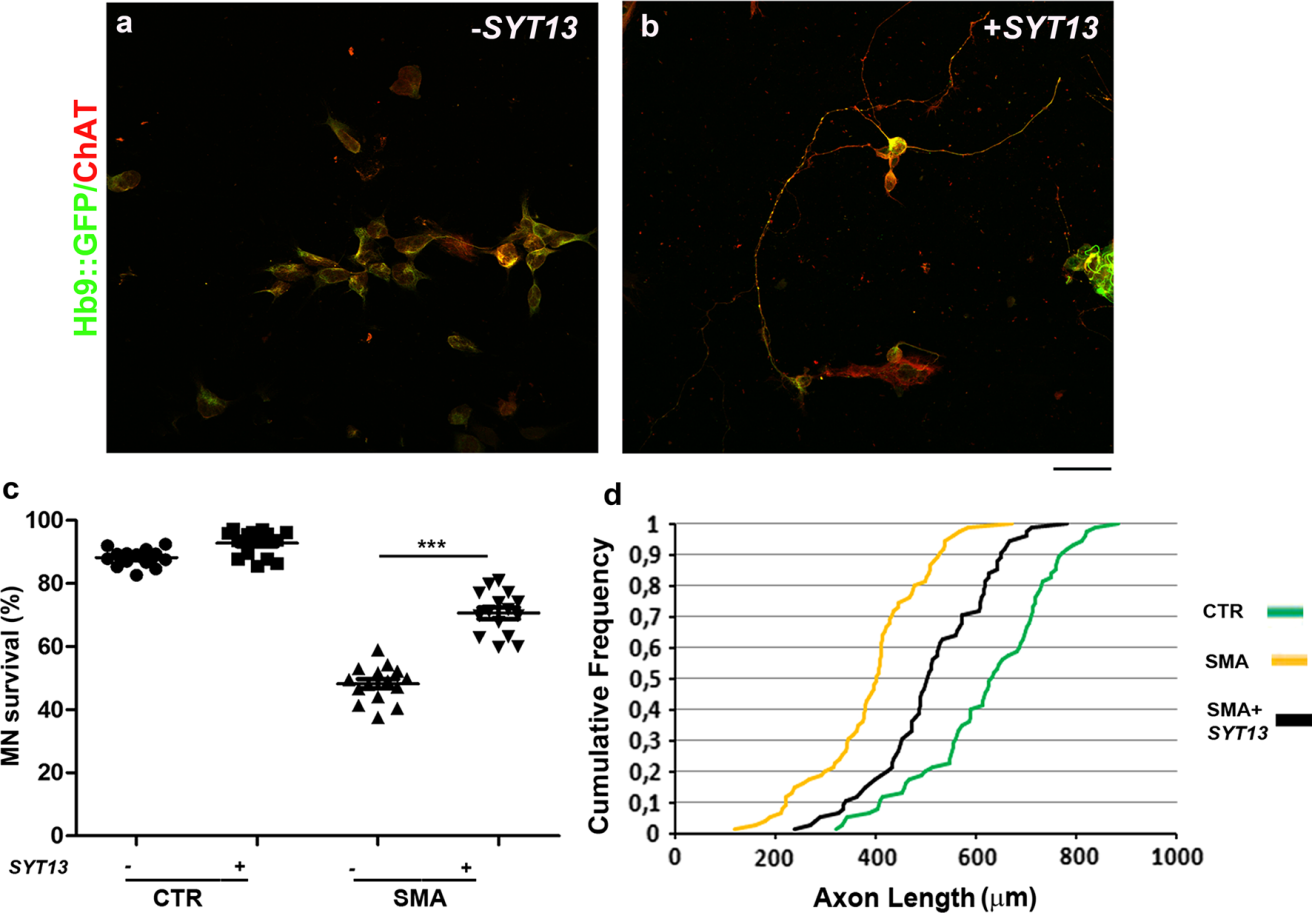
SYT13 protects human spinal motor neurons from SMA degeneration in culture. **a, b** Representative images of SMA spinal motor neurons (MNs) (Hb9::GFP, green; ChAT, red) with (**b**) and without (**a**) *SYT13* infection. Scale bar = 75 µm. **c** The number of SMA motor neurons in long-term culture was significantly diminished relative to control cells 8 weeks after differentiation. Overexpression of SYT13 was protective to motor neurons (***P < 0.0001, *F*(3,56) = 236.4; one-way ANOVA). Values are presented as means ± SEM from five independent experiments, three samples/group. **d** At eight weeks, untreated SMA motor neurons had shorter axon lengths than control cells. SMA motor neurons overexpressing SYT13 had longer axons than *null* SMA motor neurons (*P* < 0.001, two samples *K*–*S* test, five independent experiments)

To test whether *SYT13* overexpression could be protective across motor neuron diseases, we also used SMA patient motor neurons, which degenerate due to the loss of functional *SMN1*. SMA motor neurons present with apparent cell-autonomous degeneration in vitro, which is evident after 8 weeks of culture [2, 13, 19]. Overexpression of *SYT13* in SMA motor neurons (mRNA: *P* < 0.001, protein: *P* < 0.05, Supplementary Fig. 2e, f, online resource) significantly improved their survival (*P* < 0.0001; Fig. 3a–c) and increased neurite length compared to *null*-treated SMA motor neurons (*P* < 0.0001; Fig. 3d). Thus, our data demonstrate that SYT13 can protect vulnerable motor neurons from degeneration across diseases.

**Fig. 4:**
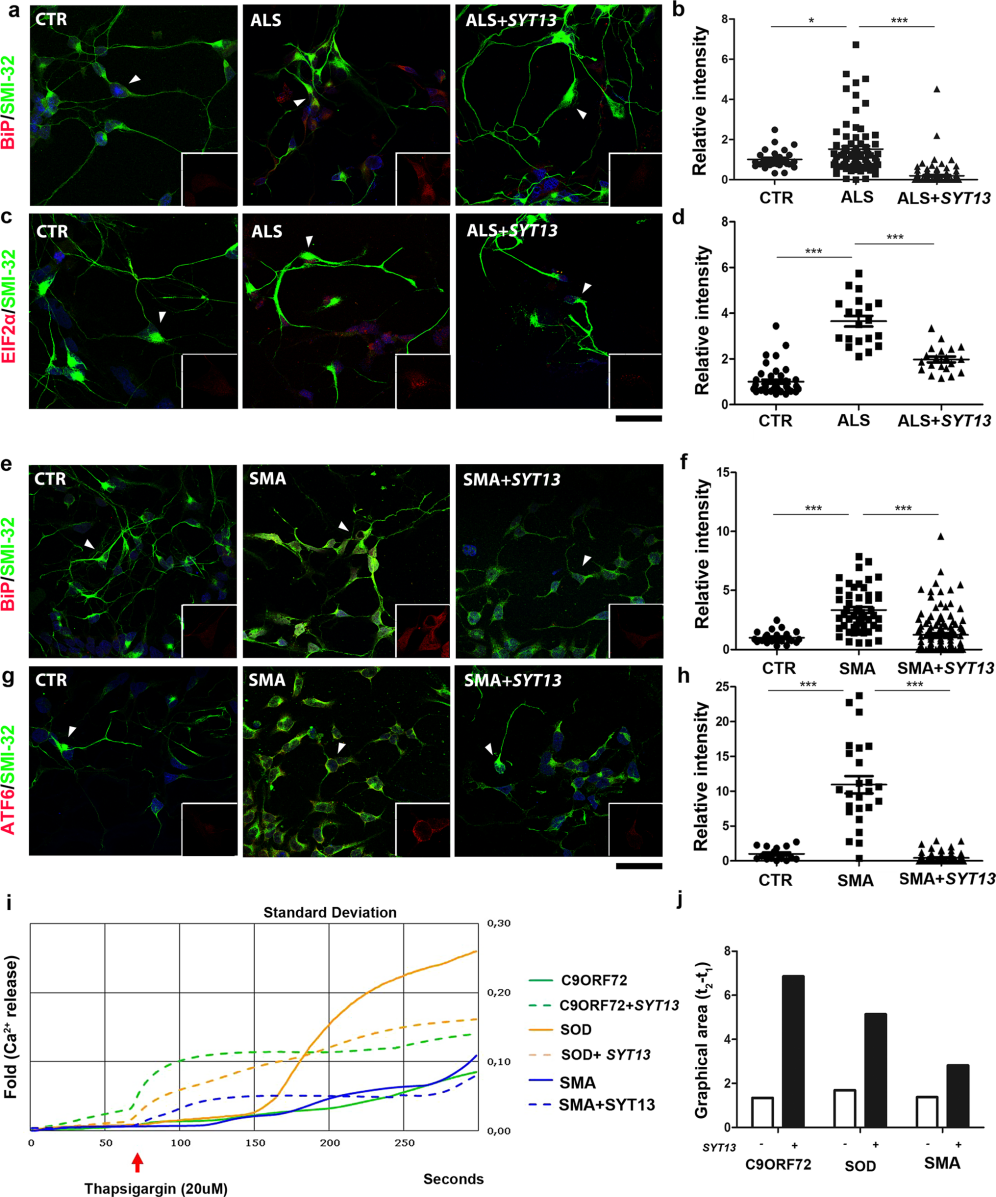
SYT13 overexpression reduces stress in ALS and SMA motor neuron cultures. **a, c** Representative images of ALS and **e, g** SMA iPSC-derived motor neurons show an increase in stress marker staining over time compared to control iPSC-motor neurons (CTR). Cells that express SYT13 had significantly reduced stress marker expression (**a, e:** BiP red and SMI32 green; **c**: pEIF2αred and SMI32 green; **g**: ATF6 red and SMI32 green). In each panel, the inset (rectangle box) shows 1.5x magnification image of stress markers signal (red) of a select region indicated by the arrow **(b, d, f, h)**. The immunoreactivity score of iPSC-derived motor neurons demonstrates that the signal of the specific stress markers was increased in ALS/SMA motor neurons with respect to CTR motor neurons and reduced in *SYT13*-treated motor neurons with respect to *null*-treated samples (*n* = 20/group, BiP ALS: *P* < 0.001; pEIF2α ALS: *P* < 0.001; BiP SMA: *P* < 0.001; ATF6 SMA: *P* < 0.001, one-way ANOVA). The immunoreactivity score was quantified using ImageJ software. Values are presented as means ± SEM. **i** Graphic showing the thapsigargin-induced calcium signal measured in C9ORF72, SOD, and SMA motor neurons with or without SYT13. SYT13 treatment restored the altered TG-evoked ER Ca^2+^release. Values are presented as means ± SD from five independent experiments, three samples/group. **l** Quantification of the area between 50 s (t1) and 150 s (t2), which is representative of the calcium signal measured in C9ORF72, SOD, and SMA motor neurons with or without SYT13

As SYT13 is implicated in vesicle trafficking, we investigated whether its up-regulation could modulate ER stress, a pathological event that occurs in both ALS and SMA [8, 44, 57, 58]. Immunofluorescence of ALS motor neuron cultures revealed a significant up-regulation of the ER stress markers, binding immunoglobulin protein (BiP) and phosphoeukaryotic initiation factor 2α (pEIF2α) compared to control motor neurons (Fig. 4a, c, middle panel, *P* < 0.05 and *P* < 0.001, respectively). SMA motor neurons also presented an up-regulation of BIP and cleaved activating transcription factor 6 (ATF6) (Fig. 4e, g, middle panel, *P* < 0.001). The overexpression of SYT13 reduced ER stress marker levels in both ALS and SMA motor neurons compared to *null*-treated motor neurons (Fig. 4a, right panel, Fig. 4b, d, *P* < 0.0001). Western blot analysis confirmed the results (Supplementary Fig. 4a, b, online resource). As activation of ER stress can lead to apoptosis, we investigated whether the observed increase in motor neuron survival after SYT13 overexpression was due to decreased apoptosis. Indeed, the activation of apoptotic protein cleaved caspase-3 (CASP3) and BAX were significantly reduced by overexpression of SYT13 in both ALS and SMA motor neurons, as shown by Western blot analysis (Supplementary Fig. 4a, b, online resource*, P* < 0.01 in ALS, *P* < 0.001 for CASP3 and *P* < 0.01 for BAX in SMA). Disruption of ER Ca^2+^ homeostasis can play a direct role in the induction of ER stress and consequent apoptosis [15, 27]. Therefore, we evaluated ER Ca^2+^ storage in ALS and SMA motor neurons before and after SYT13 overexpression. We treated motor neurons with 20 µM thapsigargin (TG) and measured the fluorescence intensity resulting from the release of ER Ca^2+^, which clearly demonstrated that SYT13 treatment restored the altered TG-evoked ER Ca^2+^ release (Fig. 4i) [15, 27]. Overall, our data suggest that SYT13 can protect human motor neurons from degeneration by mitigating pathological hallmarks of ALS and SMA, including ER stress, apoptosis and Ca^2+^ dysregulation.

### *Syt13* prolongs the lifespan of SOD1^G93A^ ALS mice by preserving motor neurons

**Fig. 5:**
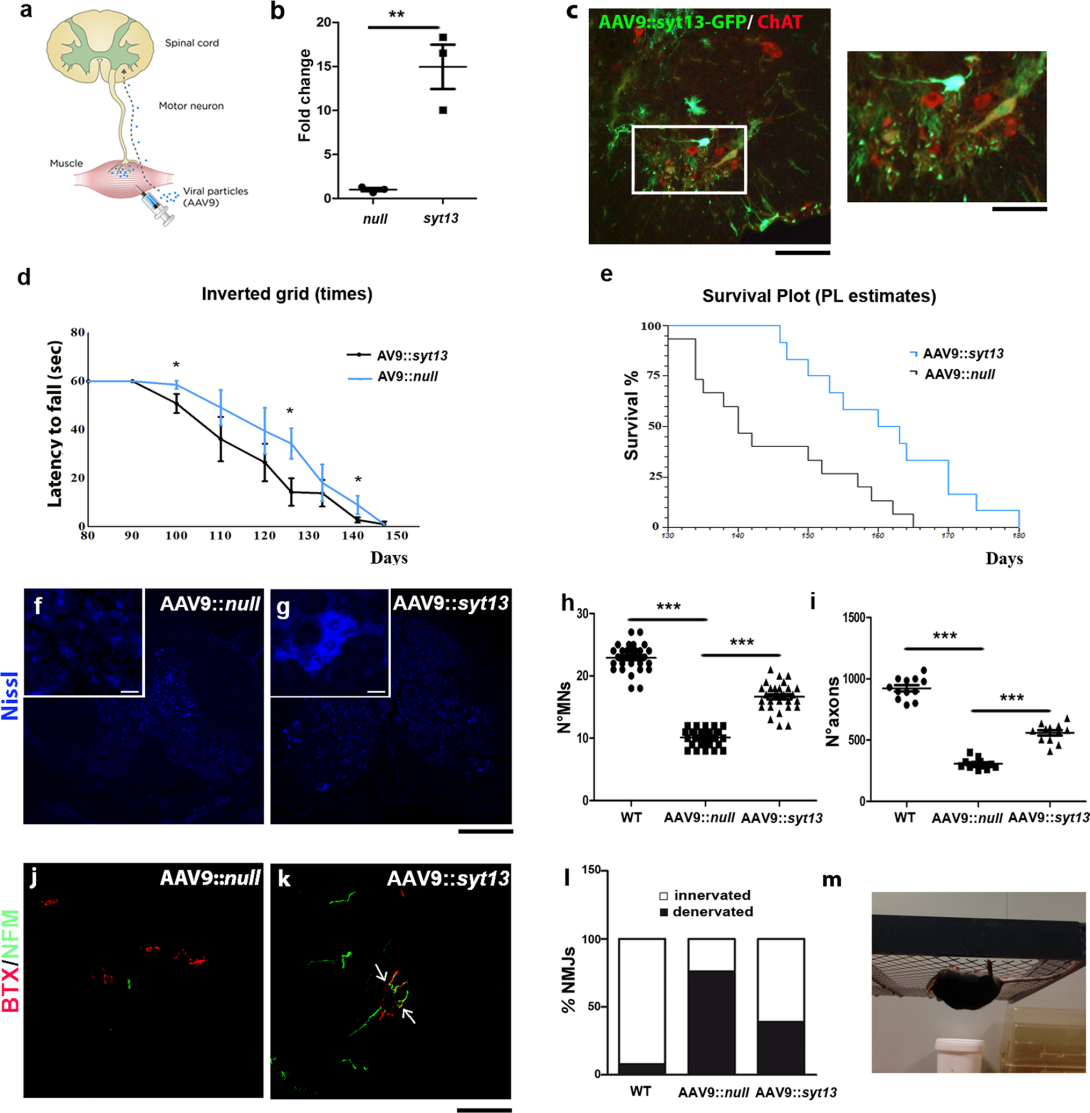
*Syt13* gene therapy extends the survival of SOD1^G93A^ ALS mice by protecting motor neurons and preserving motor axons and neuromuscular junctions. **a** Schematic drawing of in vivo* Syt13* delivery. SOD1^G93A^ mice were injected with AAV9::*GFP*, AAV9::*Syt13,* or AAV9::*null* at a total dose of 11 × 10^11^ vg bilaterally in the hindlimb quadriceps and thoracic muscles at 80 days of age (early symptomatic stage). **b** Increased levels of *Syt13* expression were detected by RT-PCR in AAV9::*Syt13* SOD1^G93A^ spinal cord versus AAV9::*null* spinal cord. **P < 0.01 (*t*(4) = 5.533, *t* test). **c** Injection of AAV9::*GFP* leads to GFP expression within the spinal cord 2 weeks post-injection. Co-localization of GFP (green) with ChAT (red) demonstrated that motor neurons were efficiently transduced (*n* = 5 mice). **d** The inverted grid performance of AAV9::*Syt13*-treated mice was significantly ameliorated with respect to AAV9*::null* mice (at P100, P126, and P141: *P* < 0.05, *n* = 6/group). Until 90 days all the mice were able to complete the test, holding the wire for 60 s, which is the baseline. **e** Kaplan–Meier survival curves demonstrates significantly extended median survival (by 20 days) in AAV9::*Syt13* treated mice (*n* = 12) compared to AAV9*::null* treated animals (*n* = 15 AAV9::*Syt13* mice, 160 ± 14 days median survival; AAV9::*null* mice 140 ± 5 days; *χ*^2^ = 8.86, *P* = 0.0029, Kaplan–Meier log rank test). **f, g** Representative images of motor neurons with Neurotracer staining (blue) in the lumbar segment of the spinal cords of AAV9*::null* (**f**) and AAV9::*Syt13* (**g**) treated mice at P120. **h, i** Quantification of motor neurons (MNs) (**h**) and axons (**i**) in the lumbar spinal cords of AAV9::*Syt13* and AAV9::*null* mice (mean ± SEM) at P120. Motor neuron and axon counts significantly increased in the AAV9::*Syt13* treated group compared to the AAV9:*null* treated group (MNs: ***P < 0.0001, *F*(2,87) = 323.96; one-way ANOVA, *n* = 30 slices counted/group, 3 mice/group; axons: ***P < 0.0001, *F*(2,33) = 224.16; *n* = 12 slices counted/group, 3 mice/group; one-way ANOVA). **l, m** Analysis of α-bungarotoxin (BTX, red) and neurofilament M (NF-M, green) in the NMJs of tibialis anterior muscles shows that AAV9::*Syt13* treatment significantly increased the number of innervated NMJs in AAV9*::Syt13 *treated SOD1^G93A^ mice (**n,** ***P < 0.0001, *χ*^2^(2) = 95.82, contingency test) compared to AAV9::*null* treated SOD1^G93A^ mice (*n* = 100 NMJs analyzed for each animal, 6 mice/group). **o** Representative image of the performance of an AAV9*::Syt13* treated SOD1^G93A^ mouse in an inverted grid test at P126. Scale bar = 75 µm in **b**, 100 μm in **f, g**, and 50 μm in **l, m**

Next, we investigated whether delivery of *Syt13* to SOD1^G93A^ ALS mice could alleviate disease pathology and prolong survival. For this purpose, an AAV9 vector was administered bilaterally into the hindlimb quadriceps and thoracic muscles in early symptomatic mice (80 days old) at a total dosage of 11 × 10^11^ particles (Fig. 5a). Injection of an AAV9::*null* vector served as the control. First, we demonstrated that an AAV9 encoding *Syt13* and *GFP* (AAV9::*Syt13*-*GFP*) was capable of extensively transducing ChAT^+^ motor neurons in the spinal cord after muscle injection (Fig. 5c). Forty-five percent of lumbar spinal motor neurons expressed GFP 2 weeks after injection. We also showed that the *Syt13*-AAV9 effectively enhanced *Syt13* expression in the spinal cords of treated mice (Fig. 5b, *P* < 0.01). AAV9::*Syt13*-treated SOD1^G93A^ mice exhibited improved neuromuscular function, as evaluated by the inverted grid test (Fig. 5d, m). Furthermore, the median survival of AAV9::*Syt13* treated mice was extended by 20 days compared to AAV9::*null* treated animals, a 14% gain (Fig. 5e, AAV9::*null* mice, 140 ± 5 days median survival; AAV9::*Syt13* mice 160 ± 14 days; *P* = 0.0029). Neuropathological analyses of spinal cords from AAV9::*Syt13* and AAV9::*null* treated mice at P120 demonstrated that the AAV9::*Syt13* treatment reduced motor neuron pathology. In particular, motor neuron loss was significantly reduced (Fig. 5f–h*, P* < 0.0001) and the axonal density in the L4 ventral root was improved (Fig. 5i, AAV9::*Syt13* mice vs. AAV9::*null* mice, *P* < 0.0001).

Next, we evaluated muscle denervation in SOD1^G93A^ mice injected with AAV9::*Syt13*. At P120, a clear preservation of innervation was demonstrated by staining against neurofilament medium and α-bungarotoxin (NF-M/BTX) in the tibialis anterior muscle of AAV9*::Syt13* treated mice (Fig. 5j-l, *P* < 0.0001). Lastly, based on our in vitro data, we evaluated any modification of the activation of ER stress and apoptosis pathways after *Syt13* overexpression in mouse spinal cords (Supplementary Fig. 5a, online resource), where we observed significantly reduced levels of ER stress and apoptotic markers.

These data demonstrate that the transfer of a single OMN-restricted gene can lead to amelioration of ALS pathological hallmarks, including motor neuron degeneration, the loss of innervation of skeletal muscles, and activation of ER stress and apoptosis.

### *Syt13* improves motor behavior and prolongs survival of SMA mice

**Fig. 6:**
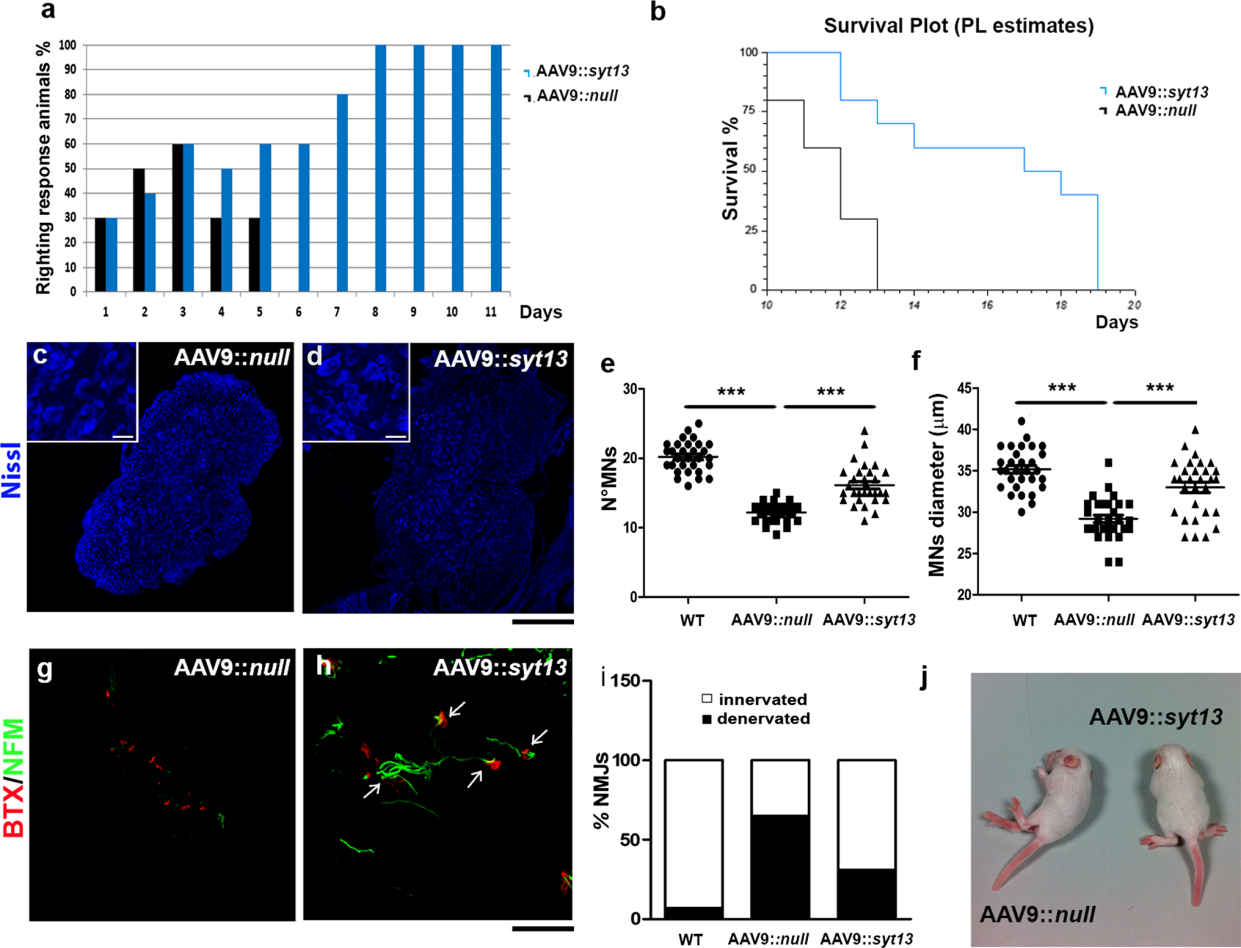
*Syt13* gene therapy prolongs the survival of SMA mice by preserving motor neurons and promoting NMJ integrity. **a** The righting performance of AAV9::*Syt13* mice improved significantly compared to AAV9*::null* mice (*P* < 0.01, *n* = 10 mice/group, logistic regression). The histogram shows the percentage of mice that can perform the test. **b** Kaplan–Meier survival curves demonstrates significantly extended survival (by 6 days) in AAV9::*Syt13* mice (*n* = 10) compared to AAV9*::null* mice (*n* = 10; *χ*^2^ = 11.25, *P* = 0.0008). **c, d** Representative motor neurons (MNs) in the lumbar segment of the spinal cords of AAV9*::null* (**c**) and AAV9::*Syt13* (**d**) mice (Nissl, blue). **e, f** Quantification of motor neurons (**e**) and their size (**f**) in the lumbar spinal cords of AAV9::*Syt13* and AAV9::*null* mice (mean ± SEM). Motor neuron number and dimension significantly increases in the AAV9::*Syt13* treatment group compared to the AAV9::*null* treated group (number: ***P < 0.0001, *F*(2,87) = 96.88, *n* = 30 slices counted/group, three mice/group; size: ***P < 0.0001, *F*(2,87) = 33.30, *n* = 30 slices counted/group, three mice/group; one-way ANOVA). **g, h** Analysis of α-bungarotoxin (BTX, red) and neurofilament M (NFM, green) in the intercostal muscles shows that AAV9::*Syt13* treatment significantly increases the number of innervated NMJs in the SMA mice compared to AAV9::*null* treatment (**i**, ***P < 0.0001, *χ*^2^(2) = 75.34, *n* = 100 NMJs, six mice/group, contingency test). (**l**) Gross appearance of an AAV9::*null* mouse, which is not able to stand on four limbs, and a AAV9::*Syt13* treated mouse at P10*.* Scale bar = 150 μm in **c, d** and 90 μm in **g, h**

Finally, we examined if the delivery of *Syt13* to transgenic SMA∆7 mice could positively affect their disease phenotype and be protective across motor neuron diseases. We injected AAV9::*Syt13* bilaterally into the hind limb quadriceps and thoracic muscles of presymptomatic SMA pups (P1) at a total dosage of 5 × 10^10^ viral particles. The overall positive effect of *Syt13* transfer was apparent from gross inspection of AAV9::*Syt13* treated SMA mice (Fig. 6l). Specifically, the mice presented with major improvements in neuromuscular function as assessed by the righting reflex, particularly after P5, when all AAV9::*null*-treated mice were unable to perform the test (Fig. 6a, *P* < 0.01). AAV9::*Syt13* delivery extended the survival of SMA mice with 6 days to a median life-span of 18 days, a 50% gain in survival compared to AAV9::*null*-treated animals (Fig. 6b, AAV9::*null* mice, 12 days median survival; AAV9::*Syt13* mice 18 days median survival; *P* = 0.0008). Quantitative neuropathological analysis at P10 revealed that AAV9::*Syt13* delivery preserved spinal motor neurons (Fig. 6c–e, *P* < 0.0001). AAV9::*Syt13* animals also presented with an increase in motor neuron soma size compared to AAV9::*null* mice (AAV9::*Syt13* mice vs. AAV9::*null* mice *P* < 0.0001, Fig. 6f), indicative of a reduced loss of large motor neurons.

Next, we analyzed muscle denervation in SMA mice treated with AAV9::*Syt13*. We detected a reduced level of denervation in the intercostal muscles at P10, as demonstrated by NF-M/BTX staining of AAV9*::Syt13* treated mice compared to AAV9::*null* treated animals (Fig. 6g–i, *P* < 0.001). Moreover, *Syt13* treatment reduced the expression of markers of ER stress and apoptosis in the spinal cords of SMA mice, as evaluated by Western blot analysis (Supplementary Fig. 5b, online resource).

Thus, *Syt13* delivery was protective across motor neuron diseases that arise from distinct genetic causes.

## Discussion

ALS and SMA are characterized by a selective loss of motor neurons, but OMNs that control extraocular muscles (EOMs) are resistant to degeneration in these fatal diseases [11, 24, 29, 32, 55]. Even stem cell-derived OMNs show an increased resilience to ALS-like toxicity compared to in vitro derived spinal motor neurons [3]. Identification of the cell intrinsic mechanisms responsible for this differential vulnerability could allow the development of therapies to prevent or slow down the progressive motor neuron loss. Notably, OMNs express several neuroprotective genes, including the calcium-regulating protein parvalbumin and insulin-like growth factor 2 (IGF-2) [10, 29, 56], which can protect mouse [29, 67] and human spinal motor neurons [2] from ALS- and SMA-like toxicity. IGF-2 could also prolong the lifespan of ALS mice by preserving motor neurons and inducing axonal regeneration [2]. In addition, GABA and glutamate receptor subunits selectively expressed in OMNs likely reduce their susceptibility to excitotoxicity [7, 10]. Moreover, Kaplan et al. (2014) demonstrated that matrix metalloproteinase-9 (MMP-9) was a vulnerability factor strongly expressed by a majority of cranial and spinal motor neurons, but almost absent from resilient OMNs and Onuf’s nuclei motor neurons. Partial reduction of MMP-9 levels in mutant SOD1 mice delayed muscle denervation and significantly extended lifespan [33]. These findings support our experimental strategy to identify therapeutic targets based on the analysis of molecular diversity between motor neuron populations that exhibit differential vulnerability to disease.

Here, we identified *SYT13* as a gene with preferential expression in OMNs compared to spinal motor neurons, and that is protective to all motor neurons in disease conditions. The preferential expression of *SYT13* in OMN somas was preserved across mice, rats and humans. Notably, SYT13 was expressed at even higher levels in OMNs from end-stage ALS patients than controls. *SYT13* was also elevated in remaining relatively resilient spinal motor neurons in end-stage ALS patient tissues compared to control, indicating that *SYT13* is either induced by the disease, or that cells with high sustained *SYT13* expression are the ones remaining at late disease stages. Future single-cell LCM-seq analysis in control and ALS patient tissues will address this matter. Collectively, our data demonstrated that relatively disease-resistant motor neurons express *SYT13*, indicating that it could have a beneficial effect on these cells.

Overexpression of SYT13 significantly protected ALS and SMA patient motor neurons from degeneration in multiple toxicity assays. Thus, the protective action of SYT13 expression seems to be independent of the etiology of degeneration. This is similar to the vulnerability factor EphA4, the suppression of which has been shown to be beneficial across ALS-causative SOD1 and TDP-43 mutations, as well as in SMN loss, with the main function being axon regeneration [68]. This across-causation functionality is highly beneficial from the perspective of therapeutic development, as mechanisms underlying motor neuron loss are largely unknown in sALS and could be different from one case to the next. Notably, we have previously, in an RNA sequencing experiment on human dopamine neurons, identified *SYT13* to be present also in this cell type [46]. Whether SYT13 is protective to dopamine neurons remains to be investigated.

SYT13 lacks a Ca^2+^-binding site, suggesting that it is more likely involved in vesicle trafficking than in synaptic regulation. Therefore, we speculate that SYT13 could also play a role in normal ER function, and that its loss could be potentially involved in ER stress. Up-regulation of ER stress is a pathological hallmark of ALS and SMA [34, 44, 57, 58] and has been strongly implicated in selective motor neuron degeneration in these diseases [34, 41, 44, 57, 58]. Interestingly, in vulnerable motor neurons, MMP-9 overexpression is involved in increased ER stress, suggesting a key role for this pathway in ALS [33]. Notably, in our experiments, *SYT13* overexpression led to the suppression of ER stress in vitro and in vivo ALS and SMA models and could restore altered TG-evoked ER Ca^2+^ release in affected motor neurons. One of the final consequences of ER stress and disruption of ER Ca^2+^ homeostasis is the induction of apoptosis. In our in vitro and in vivo ALS and SMA disease models, we observed an elevation of apoptotic hallmarks, and these levels were reduced by SYT13 overexpression. The reduction of ER stress, altered Ca^2+^ homeostasis, and apoptosis after SYT13 overexpression clearly demonstrate the beneficial effect of this gene therapy. In addition to the improvement in cell survival, SYT13 expression had a beneficial effect on motor axon length in SMA patient cells. It is plausible that SYT13 participates in axonal development, neurite outgrowth, and axonal repair similar to SYT1 and SNAP25 [23, 26, 51], but this remains to be further investigated.

*Syt13* gene therapy extended the survival of ALS (SOD1^G93A^) and SMA (SMAΔ7) mice by preserving motor neuron somas, axons, and the innervation of muscle endplates. The preservation of innervation is likely the underlying reason for the improved neuromuscular function and survival observed in treated animals. The protection of NMJs was more pronounced than the cell soma survival in vivo*,* and increased axon length was detected in vitro in SMA patient motor neurons, suggesting that Syt13 expression may act on axonal regeneration in vivo, similar to IGF-2 [2]. We delivered *Syt13* at a time point when NMJs are already starting to be denervated in SOD1^G93A^ mice [10]. Therefore, we speculate that Syt13 could be beneficial to motor neurons in symptomatic disease phases when more motor axons have started to retract from the muscle.

Our results suggest *Syt13* as a promising candidate for future therapeutic interventions in motor neuron diseases. Syt13 overexpression improved motor neuron survival and delayed muscle denervation in ALS and SMA mice, leading to a 14% increase in lifespan of ALS mice and a 50% increase in SMA mice. Even a small increase in Syt13 expression was sufficient to offer substantial benefits. Interestingly, Syt13 appears to act independent of the cause of disease in sALS, fALS, and SMA.

The current absence of effective therapies in ALS reflects the small number of therapeutic targets with significant efficacy in in vivo models other than modulation of the causative mutant *SOD1* [9, 20]. The recently approved drugs for SMA, the antisense oligonucleotide Nusinersen and the gene therapy with AAV9-SMN1 [22, 46], represent a remarkable turning point for SMA therapy, with promising and exciting results. However, the beneficial effects can vary based on several factors, particularly the timing of treatment and disease severity. It is a well-documented fact that it is crucial to supplement SMN as early as possible in the treatment of SMA [25]. In addition, it is possible that increasing only SMN may not completely address the slow neurodegenerative process that causes progressive functional decline beyond childhood in less severe SMA types [6]. Furthermore, patients treated with SMN-based therapy may simply present delayed symptoms instead of rescued symptoms if recovery of the neuromuscular system is incomplete. Thus, finding SMN complementary/independent or downstream targets responsible for selective motor neuron dysfunction can be important for a comprehensive whole-lifespan therapeutic approach that comprises symptomatic cases and all SMA clinical phenotypes. In this context, the neuroprotective effect of SYT13 might warrant further exploration at the preclinical level in combination with SMN-based therapies. The most rational approach for genetic ALS forms appears to be gene therapy correction, particularly the selective reduction of toxic RNAs and/or proteins [49, 59]. However, the vast majority of ALS cases are sporadic and of unknown origin. Thus, finding a gene therapy approach to protect motor neurons by modulating gene expression of target genes, such as *SYT13*, is highly relevant from a therapeutic point of view in both sporadic and familial cases.

Overall, our analysis demonstrates that deducing the biological diversity of neuronal subpopulations can identify neuroprotective pathways for effective therapies in ALS and SMA, revealing *SYT13* as a protective gene for motor neurons. Moreover, our strategy focused on genetic modification of neuronal subtypes displaying selective vulnerability may also have an impact in neurodegenerative diseases affecting other neuron classes.

## Electronic supplementary material

Below is the link to the electronic supplementary material.

Supplementary file1 (DOC 6091 kb)
