# Supplementary material for: Synaptotagmin 13 is neuroprotective across motor neuron diseases
Source: Acta Neuropathol. 2020 Feb 17;139(5):837–53. doi: 10.1007/s00401-020-02133-x (PMC7181443; doi:10.1007/s00401-020-02133-x)
Supplement: Supplementary file 1 — Supplementary file1 (DOC 6091 kb) [file 401_2020_2133_MOESM1_ESM.doc]

**Supplementary Tables**

**Table S1 Characteristics of non-demented and ALS clinical cas**es

| **Case** | **Sex** | **Age** | **Cause of death** | **Postmortem time(h)** | **Source** | **Analysis** |
| --- | --- | --- | --- | --- | --- | --- |
| 1 | F | 47 | Respiratory failure, metastasis | 4,0 | NBB | ISH |
| 2 | M | 51 | Suicide | 7,8 | NBB | ISH |
| 3 | M | 58 | Thrombosis | 5,3 | NBB | ISH, LCM |
| 4 | F | 58 | Colon cancer | 14,1 | NDRI | IHC |
| 5 | M | 59 | Unknown | 4,4 | NBB | ISH, LCM |
| 6 | M | 63 | Respiratory arrest | 2,2 | NDRI | LCM |
| 7 | F | 68 | Obstructive pulmonary disorder | 3,0 | NDRI | LCM |
| 8 | M | 71 | Sepsis | 7,4 | NBB | IHC |
| 9 | F | 71 | Kidney failure | 7,1 | NBB | IHC |
| 10 | F | 71 | Renal insufficiency | 7,3 | NBB | ISH |
| 11 | F | 72 | Metastized bile duct cancer | 7,5 | NDRI | LCM |
| 12 | M | 79 | Bronchopneumonia and sepsis | 7,7 | NBB | LCM |
| 13 | F | 79 | Respiratory arrest | 13,3 | NDRI | LCM |
| 14 | M | 82 | Pleuritis carcinomatosis, cachexia | 10,0 | NBB | LCM |
| 15 | F | 84 | Unknown | 6,9 | NBB | ISH |
| 16 | F | 88 | Metastized lung cancer | 10,0 | NDRI | LCM |
| 17 | M | 88 | Metastized prostate cancer | 7,4 | NBB | LCM |
| 18 | M | 98 | Atherosclerotic aorta | 8,7 | NBB | LCM |
| 19 | F | 99 | Unknown | 4,3 | NBB | LCM |
| 20 | M | 33 | ALS, euthanasia | 5,9 | NBB | LCM |
| 21 | M | 56 | ALS | 9,5 | NDRI | LCM |
| 22 | F | 57 | ALS, respiratory failure | 23 | NDRI | LCM |
| 23 | M | 59 | ALS | 17,5 | NDRI | LCM |
| 24 | M | 62 | ALS | 6,9 | NBB | LCM |
| 25 | F | 68 | ALS | 5,0 | NBB | LCM |
| 26 | M | 70 | ALS | 13,8 | NDRI | LCM |
| 27 | M | 71 | ALS | 6,8 | NBB | LCM |
|  |  |  |  |  |  |  |

NBB- Netherland's Brain Bank(http://www.brainbank.nl)

NDRI- National Disease Research Interchange (<https://ndriresource.org/)>

ISH- in situ hibridization (Single molecule-RNA scope)

LCM- Laser Capture Microdissection for RNA sequencing

IHC- Immunohistochemistry

**Table S2**. **Characteristics of human fibroblast-derived iPSC lines**

| **iPSCline** | **Diagnosis** | **Sex** | **Age** | **Reprogrammingstrategy** |
| --- | --- | --- | --- | --- |
| 27b | ALS(SOD1/G85S) | Female | 29 | Retrovirus, 3factors:OSK |
| 29d | ALS(SOD1/L144F) | Female | 82 | Retrovirus 4factors:OSKM |
| AL/ALS1.1 | ALS(SOD1/L144F) | Female | 55 | Non viral 6factors:OSKM+LN |
| SC603A/B-ALS | ALS,sporadic | Female | 61 | Retrovirus, 4factors:OSKM |
| AM/ALS1.1 | ALS,sporadic | Female | 77 | Non viral 6factors:OSKM+LN |
| SMA1.1 | SMAI | Male | 3 | Non viral 6factors:OSKM+LN |
| SMA2.1 | SMAI | Male | 2 | Non viral 6factors:OSKM+LN |
| 19.9 | HealthyDonor | Male | newborn | Non viral 6factors:OSKM+LN |
| CP13c | HealthyDonor | Female | 45 | Non viral 6factors:OSKM+LN |
| iPSForeskin | HealthyDonor | Male | newborn | Lentivirus, 4factors:OSNL |
| 11b | HealthyDonor | Male | 36 | Retrovirus 3factors:OSK |
| 15b | HealthyDonor | Female | 48 | Retrovirus 3factors:OSK |
| 1C9ALS | C9 exp | Female | 44 | Non viral 6factors:OSKM+LN |
| 2C9ALS | C9 exp | Female | 47 | Non viral 6factors:OSKM+LN |

**Table S3. Antibodies used for immunohistochemistry, immunocytochemistry and w**estern blot

| **Target** | **Source** | **Host species** | | **Concentration used** | | | |
| --- | --- | --- | --- | --- | --- | --- | --- |
|  |  |  | **mouse tissue** | | **human tissue** | ***in vitro*** | **western blot** |
| **ChAT** | Millipore | Rabbit | 1:200 | | - | 1:125 | - |
| **SYT13** | Proteintech | Rabbit | 1:20 | | 1:100 | 1:25 | 1:1300 |
| **SYT13** | Atlas Ab | Rabbit | 1:500 | | - | - | 1:500 |
| **BiP** | Abcam | Rabbit | - | | - | 1:400 | 1:1000 |
| **Cl. Caspase-3** | Cell Signaling | Rabbit | - | | - | 1:100 | 1:1000 |
| **Bax** | Cell Signaling | Rabbit | - | | - | 1:100 | 1:1000 |
| **ATF6** | Abcam | Rabbit | - | | - | 1:1000 | 1:500 |
| **pEIF2α**  **β-actin** | Millipore  Sigma | Rabbit  Rabbit | -  - | | -  - | 1:200  - | 1:1000  1:1000 |
| **SMI32**  **HB9**  **NFM**  **α-bungarotoxin**  **Alexa555** | Covance  Abcam  Millipore  Life Tech | Mouse  Rabbit  Rabbit | -  -  1:250  1:200 | | -  -  -  - | 1:1000  1:200  -  - | -  -  -  - |

**Supplementary Figures**

**Figure S1**

**
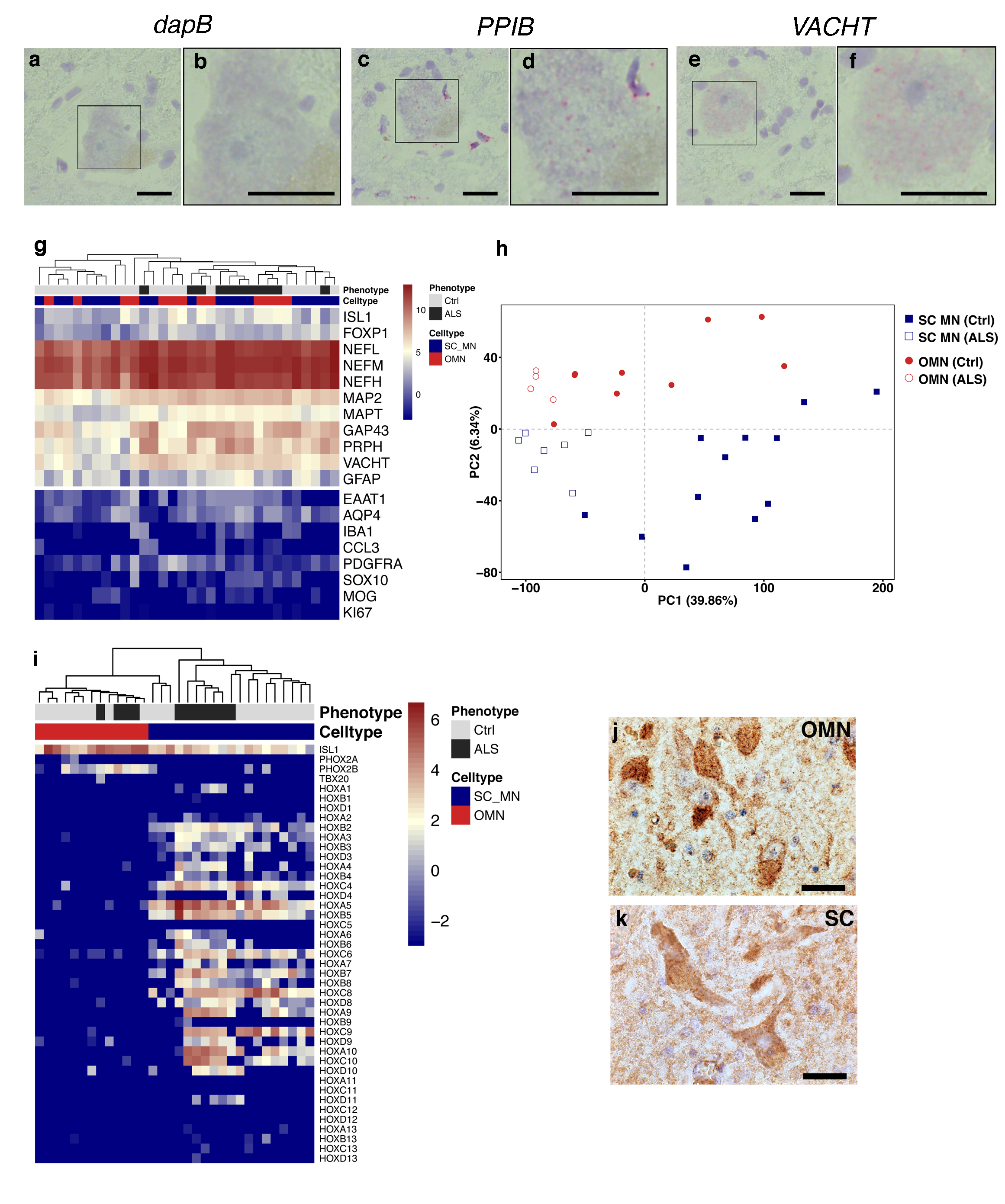
**

**Fig. S1. RNA scope validation, RNA seq and Syt13 IHC in human tissues**

One negative control probe (*dapB*-C1, **a-b**) and two positive control probes were tested for the RNA scope procedure, including a probe against (**c-d**) peptidylprolyl isomerase b (*PPIB*-C1) and one against (**e-f**) the vesicular acetylcholine transporter-member A3 (*VACHT*-C1). (**g**) Quality analysis of RNA sequencing data on neurons isolated from human *post mortem* tissues showed that neuronal markers including neurofilaments, tubulins, peripherin and VACHT were expressed at high levels across OMN and spinal MN samples. Glial markers were present at significantly lower levels and markers of oligodendrocytes and microglia were absent in the majority of samples. (**h**) PCA of all human LCM-seq samples based on all genes expressed at >1 RPKM across N=5 samples or more. (**i**) Analysis of Hox and Phox gene expression, which clustered OMNs away from spinal motor neurons based on their distinct anterior posterior positions in the nervous system. (**j-k**) Immunohistochemistry in human control *post mortem* tissues with an antibody against SYT13 showed the presence of SYT13 protein in (**f**) OMNs and (**g**) spinal MNs. Scale bars in **a-f** and **j-k** all represent 30 μM in length.

**Figure S2**

**
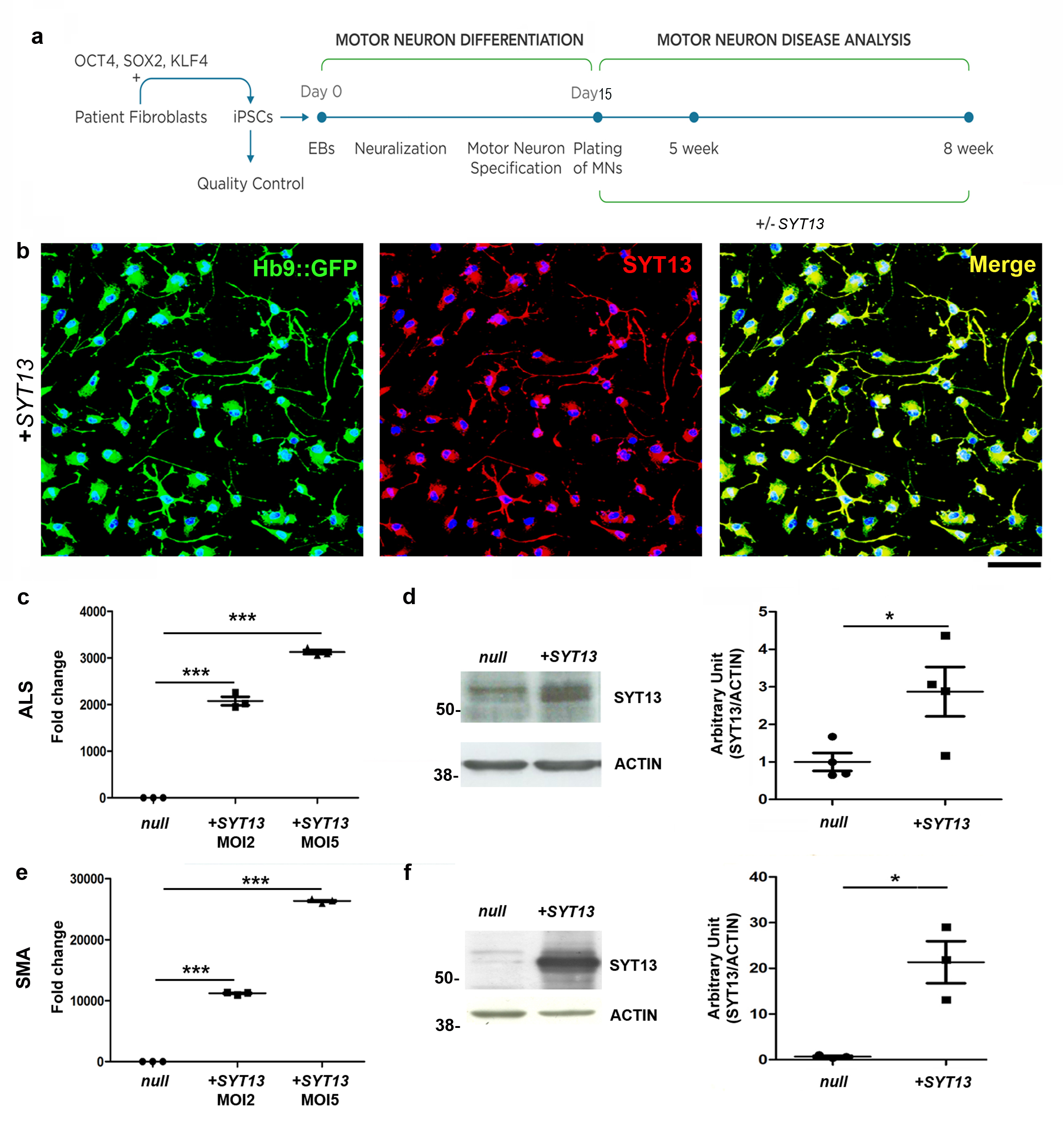
**

**Fig. S2. Differentiation and genetically modification of human iPSCs derived MNs**

(**a**) Schematic illustration of experimental design: timing of MN differentiation and their genetic modification to express SYT13. (**b**) Representative image of differentiated iPSCs-MNs*SYT13*infected, labelled with GFP reporter under the Hb9-MN specific promoter (green) and SYT13 (red). DAPI, blue signal Scale bars: **b:** 100µm. (**c,d**) After transduction, human ALS spinal MNs obtained from iPSCs significantly increased expression of SYT13 compared to cells transfected with the *null* vector as detected by RT-PCR (MOI2:****P<*0.001; t(4)=22,72; t-test ; MOI5:****P<*0.001; t(4)=67,76; t-test)and by western blot (**P*<0.05; t(6)=2.677; t-test). (**e,f**) After transduction, human SMA spinal MNs significantly increased expression of SYT13 compared to cells transfected with the *null* vector as detected by RT-PCR (MOI2: ****P<*0.001; t(4)=85.03; t-test; MOI5:****P<*0.001; t(4)=136.8; t-test) and by western blot analysis (***P*<0.01; t(4)=4.489; t-test). Values show means ± SEM from 3 independent experiments.

**Figure S3**

**
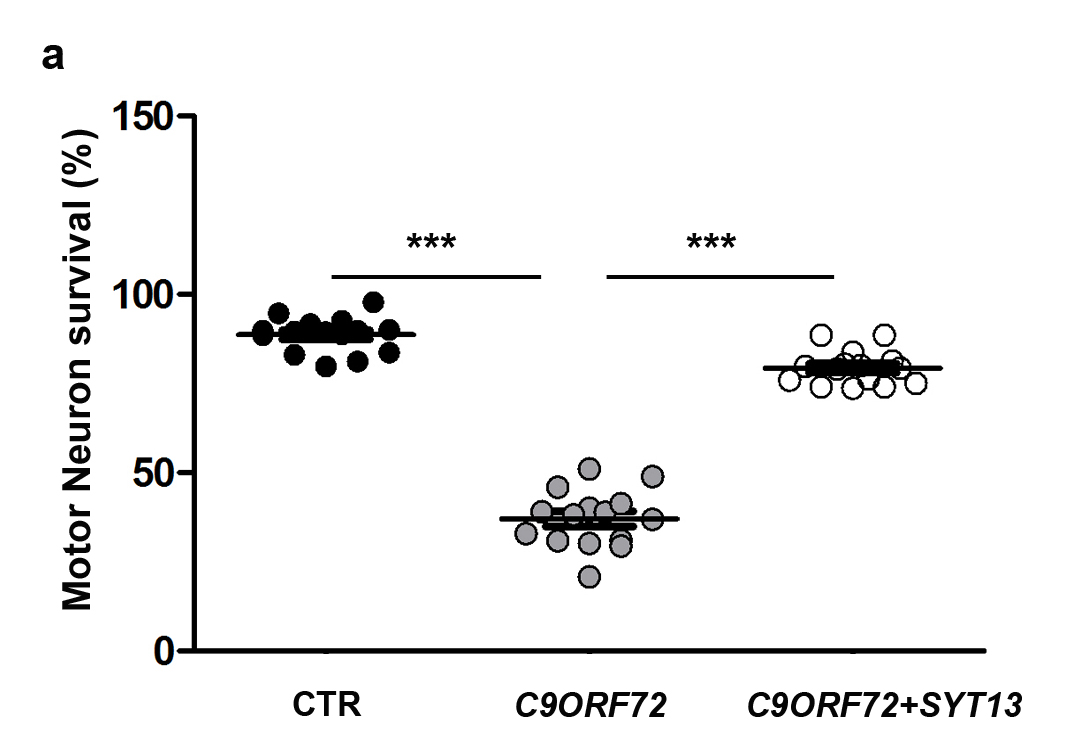
**

**Fig. S3. SYT13 overexpression increases the survival of ALS-C9ORF72 MNs in cultures**

The ALS-C9ORF72 motor neurons presented a cell-autonoumous death in culture after 3 weeks (****P*<0.001 one-way ANOVA), which was rescued after SYT13 treatment (*P*<0.001 one-way ANOVA).Values show means ± SEM from 3 independent experiments.

**Figure S4**

**
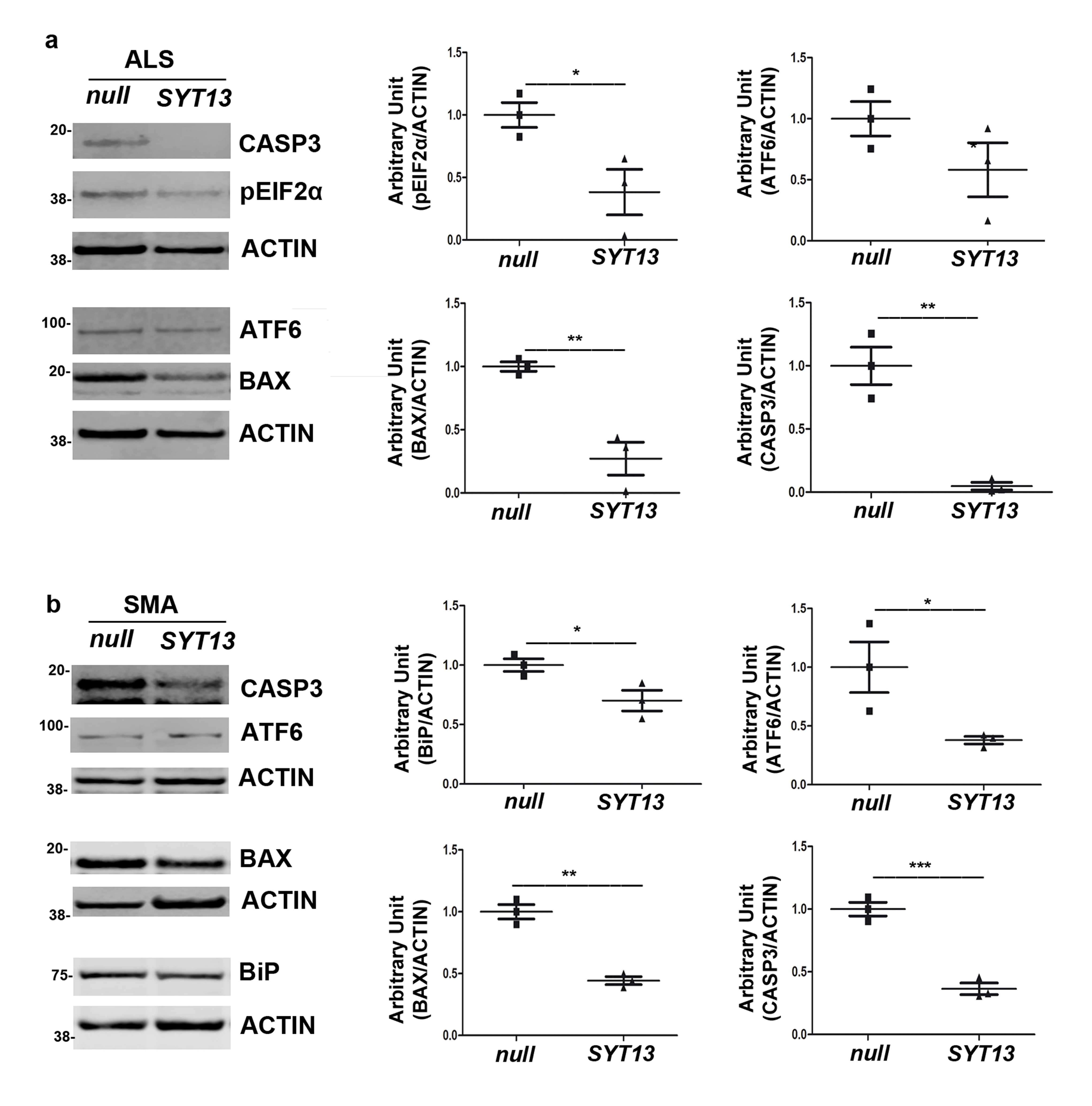
**

**Fig. S4. SYT13 overexpression reduces ER-stress and apoptosis in ALS and SMA MN cultures**

(**a**) After SYT13 overexpression, ALS spinal MNs showed a reduction in pEIF2a (**P<*0.05; t(4)=2.969; t-test), ATF6, BAX (***P<*0.01; t(4)= 5.398; t-test) and CASP3 (***P<*0.01; t(4)=6.299; t-test) expression compared to *null* treated ALS MNs as detected by western blot. (**b**) After SYT13 overexpression, SMA spinal MNs showed a significantly reduction in BiP (**P<*0.05; t(4)=2.940; t-test), ATF6 (**P<*0.05; t(4)=2.855; t-test), BAX (***P<*0.01; t(4)=8.407; t-test) and CASP3 (****P<*0.001; t(4)=8.869; t-test) expression compared to untreated SMAMNs as detected by western blot. Values show means ± SEM from 3 independent experiments.

**Figure S5**

**
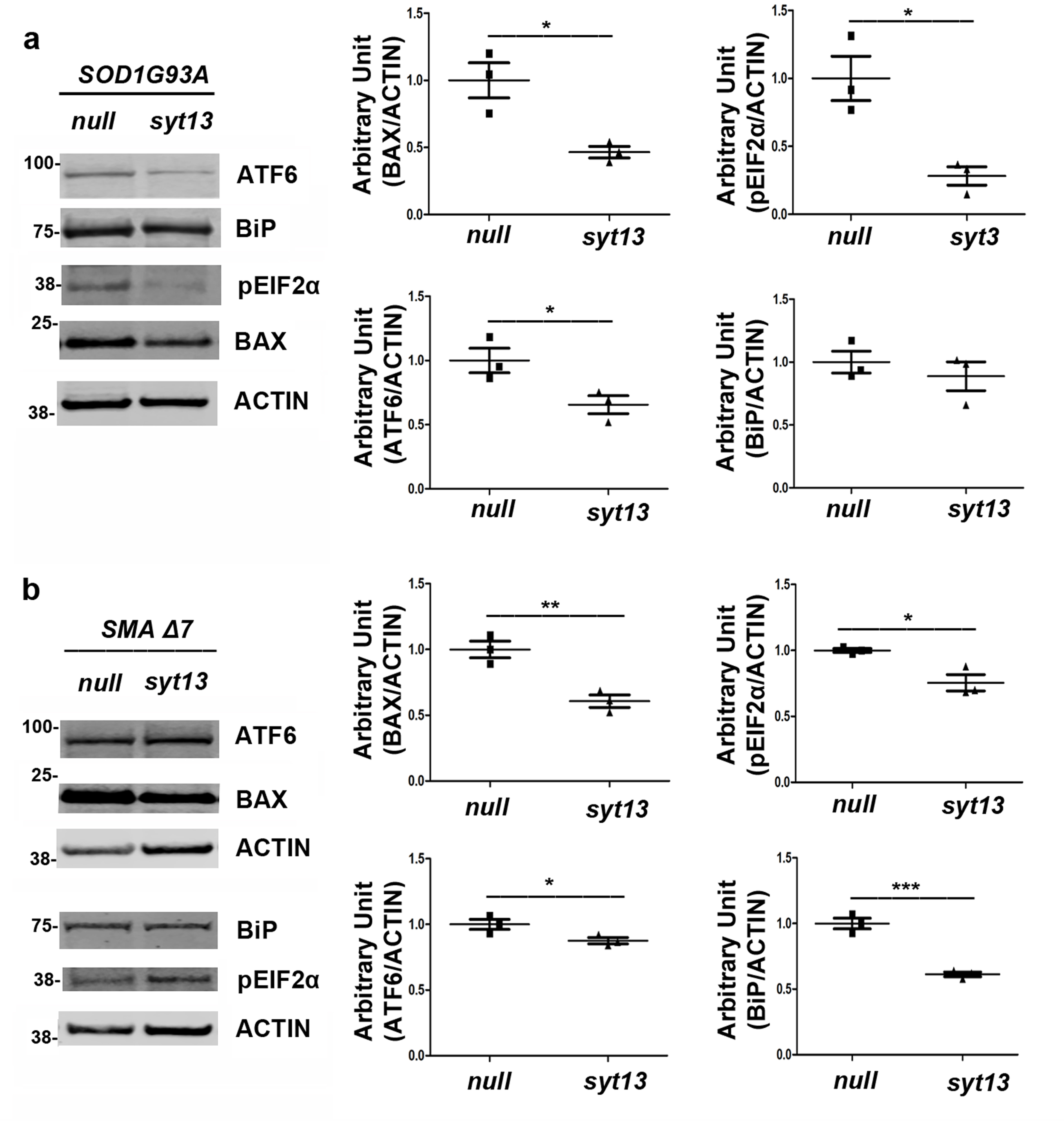
**

**Fig. S5. SYT13 overexpression reduces ER-stress and apoptosis in SOD1G93A and SMAΔ7 spinal cord**

(**a**) After AAV9::*Syt13* overexpression, BAX (**P<*0.05;t(4)=3.881; t-test), pEIF2a (**P<*0.05; t(4)=4.076; t-test), ATF6 (**P<*0.05; t(4)=2.908; t-test) and BiP expression was reduced in SOD1G93A spinal cord compared to AAV9*::null* ones, as detected by western blot. (**b**) After AAV9::*Syt13* overexpression, SMAΔ7 spinal cord showed a reduction in BAX (***P<*0.01; t(4)=5.000; t-test), pEIF2a (**P<*0.05; t(4)=3.864; t-test), ATF6(**P<*0.05;t(4)=2.787; t-test), and BiP (****P<*0.001; t(4)=8.718; t-test)expression, as detected by western blot. Values show means ± SEM.
